# Clinical Profiles, Disease Outcome and Co-Morbidities among *T. b. rhodesiense* Sleeping Sickness Patients in Uganda

**DOI:** 10.1371/journal.pone.0118370

**Published:** 2015-02-26

**Authors:** Charles D. Kato, Ann Nanteza, Claire Mugasa, Andrew Edyelu, Enock Matovu, Vincent P. Alibu

**Affiliations:** 1 School of Bio-security, Biotechnical & Laboratory Sciences, College of Veterinary Medicine, Animal Resources & Bio-security, Makerere University, Kampala Uganda; 2 College of Natural Sciences, Makerere University, Kampala Uganda; 3 Lwala Hospital, Kaberamaido Uganda; Brighton and Sussex Medical School, UNITED KINGDOM

## Abstract

**Background:**

The acute form of Human African Trypanosomiasis (HAT, also known as Sleeping sickness) caused by *Trypanosoma brucei rhodesiense* has been shown to have a wide spectrum of focus specific clinical presentation and severity in East and Southern Africa. Indeed HAT occurs in regions endemic for other tropical diseases, however data on how these co-morbidities might complicate the clinical picture and affect disease outcome remains largely scanty. We here describe the clinical presentation, presence of co-infections, and how the latter impact on HAT prognosis.

**Methods and Findings:**

We carried out a retrospective analysis of clinical data from 258 sleeping sickness patients reporting to Lwala hospital between 2005 and 2012. The mean patient age was 28.6 years with a significant number of cases below 18 years (p< 0.0001). About 93.4% of the cases were diagnosed as late stage (p< 0.0001). The case fatality rate was 10.5% with post treatment reactive encephalopathys reported in 7.9% of the cases, of whom 36.8% eventually died. Fever was significantly (p = 0.045) higher in patients under 18 years. Of the early stage patients, 26.7% and 6.7% presented with late stage signs of sleep disorder and mental confusion respectively. Among the co-infections, malaria was significantly more prevalent (28.9%; p< 0.0001) followed by urinary tract infections (4.2%). Co-infections were present in 14.3% of in-hospital deaths, 38.5% of which were recorded as Malaria. Malaria was significantly more common in patients under 18 years (45.5%; p< 0.02), and was reported in 60% of the fatal cases in this age group.

**Conclusions:**

We show a wide spectrum of sleeping sickness clinical presentation and disease outcome that was apparently not significantly influenced by concurrent infections. It would thus be interesting to determine the host and/or parasite factors that might be responsible for the observed diverse clinical presentation.

## Introduction

Human African Trypanosomiasis (HAT) or sleeping sickness is caused by extra-cellular protozoan parasites *T*. *b*. *rhodesiense* (East and Southern Africa) and *T*. *b*. *gambiense* (West and central Africa). It is considered that each species produces a different disease. *Trypanosoma brucei rhodesiense* HAT has been described as acute while *T*. *b*. *gambiense* HAT takes a chronic course [1]. Tsetse fly vectors of the genus *Glossina* that is restricted to sub-Saharan Africa transmit both diseases. Although the number of new cases in the region is decreasing, an estimated 12.3 million people are at a risk of acquiring the disease [2,3]

HAT progresses in two stages, the hemo-lymphatic or early stage is characterized by the proliferation of trypanosomes in blood, lymph and other body tissues. The second or late stage appears after weeks in *T*. *b*. *rhodesiense* disease or months in *T*. *b*. *gambiense*, and is characterized by invasion of trypanosomes in the central nervous system (CNS). A wide spectrum of clinical presentation of *T*. *b*. *rhodesiense* HAT in East Africa was previously reported [4,5]. A study by MacLean et al. [5] in Uganda while comparing patients in geographically similar areas (Tororo and Soroti) reported differences in disease presentation and progression. The possible contribution of co-infections to the observed profiles was however not investigated.

Sleeping sickness due to *T*. *b*. *rhodesiense* manifests as an acute disease, with death occurring within weeks or months [6]. The Chancre is the first sign of this disease in 5–26% of the patients [3,7], as an immediate inflammatory response to the inoculated trypanosomes after a tsetse fly bite. This is followed by non-specific early stage signs like fever, malaise, headache, pruritus, transient edema, lymphadenopathy and splenomegaly [4]. The late stage is characterized by disturbances in the sleep cycle, headache, psychological and behavioral changes, tremors, motor weakness, sensory disturbances, poor coordination, loss of consciousness, coma and eventually death [8].

HAT is fatal if untreated. Early stage *T*. *b*. *rhodesiense* disease is treated with intravenous (IV) suramin, but the drug may cause complications such as renal failure, skin lesions, anaphylactic shock and peripheral neuropathy [8,9]. The trivalent organic arsenical melarsoprol is the only drug used to treat late stage *T*. *b*. *rhodesiense*. Melarsoprol is toxic and causes reactive encephalopathy (RE) in up to 10% of the patients leading to about 5% fatality [10]. Risk factors to reactive encephalopathy include the presence of trypanosomes in cerebrospinal fluid (CSF) and a CSF white cell count (WCC)>100 cells/mm^3^[10]. Treatment failure due to melarsoprol has also been reported [11,12]

The impact of co-infections on the outcome of neglected tropical diseases necessitates further research and has been recommended as a priority area to the European Union [13]. However, literature about co-infections among HAT patients as regards prevalence, effect on clinical presentation and disease outcome is limited. Such information would be of importance in early disease diagnosis and provide a basis for better treatment regimen for co-infected patients so as to improve disease prognosis. We describe here, the clinical presentation, co-infections and disease outcome of 258 *T*. *b*. *rhodesiense* HAT patients presented at Lwala hospital in North Eastern Uganda between 2005 and 2012. Further we seek to determine the impact that co-infections have on HAT prognosis.

## Materials and Methods

### Ethical statement

Ethical review of this study was by the Institutional Review Board (IRB) of the Vector Control Division, Ministry of Health; final approval was provided by the Uganda National Council for Science and Technology (UNCST). This was a retrospective study in which all data analyzed was recovered from that routinely collected as a requirement for HAT diagnosis and treatment following national guidelines. HIV testing was done on a subset of participants for whom it was deemed necessary by the responsible clinician, following ministry of health guidelines. For purposes of this study all the data was anonymized prior to analysis.

### Study site and study design

**Fig 1:**
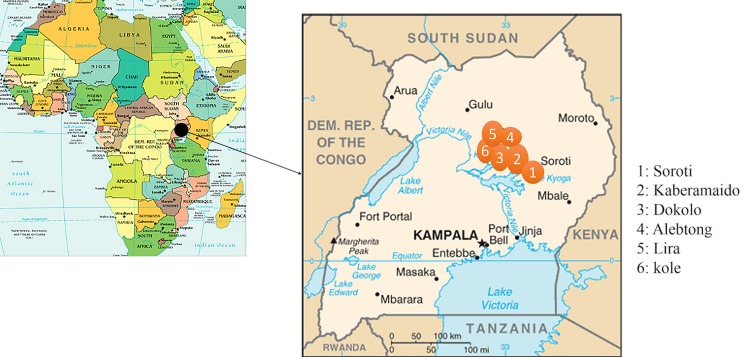
Map of Uganda showing the six (6) districts from which sleeping sickness patients were recruited. Maps were sourced from the Central Intelligence Agency. https://www.cia.gov/library/publications/the-world-factbook/docs/refmaps.html.

Lwala hospital is a sleeping sickness referral center in North Eastern Uganda (Kaberamaido district). The hospital serves a large catchment area spanning several districts including Kaberamaido, Dokolo, Alebtong, Kole, Lira and Soroti (Fig. 1). Between 2005 and 2012, five hundred seventy one (571) HAT patients presented at the hospital. The majority of cases were diagnosed at the hospital; few were referred by field surveillance teams or peripheral health centers. For this retrospective study, 258 patients had complete medical records and were reviewed. The routine diagnosis of suspected HAT patients, was done by microscopic examination of wet and thick blood films from finger prick blood [14]. If trypanosomes were present in the blood smear, or the patient presented with highly suspicious HAT signs, a lumbar puncture was performed following WHO [3] disease staging guidelines. Analysis of cerebrospinal fluid for trypanosomes and White blood cell (WBC) counts was done microscopically using the Neubauer Haemocytometer method. Late stage infection was confirmed by the presence of trypanosomes in the CSF and/or a White blood cell count of ≥ 5 cells/mm^3^. Hemoglobin (HB) levels were measured from peripheral blood by calculating hematocrit levels from packed cell volume. Patients with HB levels of ≤ 8g/dL were recommended for blood transfusion. Treatment of all HAT patients was carried out following the recommendations of the national sleeping sickness control program; early stage patients were treated with intravenous Suramin while late stage patients received an initial dose of Suramin followed by a daily dose of melarsoprol.

### Clinical examination

Upon arrival at the hospital, a detailed clinical history was sought from each patient. For terminally ill patients, clinical history was provided by the care taker. Disease duration was taken as the period when the patient reported the first HAT related clinical sign. Patients were physically examined by a medical officer, clinical officer or an experienced nursing officer. Reactive encephalopathy was diagnosed in patients with rapid worsening of neurological symptoms, psychotic reactions, convulsions and progressive coma. Data recorded on the clinical record form included demographic characteristic, self-reported symptoms, perceived onset of symptoms, clinical presentation of the disease, laboratory findings, treatment schedule and disease outcome. Neurological involvement was recorded such as convulsions, tremors, urinary incontinence, psychotic behavior and sleep disorders.

### Co-infection assessment

Co-infections were diagnosed as described by Cheesebrough [15]. Typhoid was screened using the Widal method, while malaria screening was done by the observation of parasites on Giemsa or Field stained thick blood films. Tuberculosis was diagnosed using sputum microscopy while Human Immunodeficiency Virus (HIV) suspected cases were voluntarily tested using rapid diagnostic tests following guidelines provided before by the Ministry of Health. Urinary tract infections (UTI) were diagnosed using a urine test strip that was compared to a colored scale. Amebiasis was diagnosed by the detection of cysts in stool using iodine stained wet smears.

### Data management and statistical analyses

Data were anonymized and entered into SPSS version 18 statistical software. Numerical variables were summarized using mean and standard error of the mean (SEM) and variables compared using t-tests set at significance level of p< 0.05. In order to determine associations between factors, univariate analysis of categorical data was done using cross-tabulation with a Chi-square or Fisher’s exact test. All variables with a p-value of ≤ 0.25 from univariate analyses and having cell counts ≥ 5 were considered as candidate variables to the multivariate analysis for sequential model building. The final model was taken to be significant when it had a p-value of ≤ 0.05

## Results

### Demographic and baseline diagnostic characteristics

A significantly high number of cases were from Kaberamaido district (165/239; 69%; p< 0.0001), followed by Dokolo district with 54 cases (22.6%). Alebtong had 13 (5.4%), Soroti 5 (2.1%), Lira and Kole each with 1 (0.4%; see Table 1). Data on district of residence for 19 patients was not recorded. Within Kaberamaido, the majority of cases were from Alwa (25.7%) and Otuboi (24.3%) sub-counties. There were more female patients 134 (51.9%) compared to males 124 (48.1%) although this difference was not significant. The mean age of patients was 28.6 years (0.1–85) with a significant number of cases below 18 years (p< 0.0001). Male cases were significantly more common among patients below 18 (p< 0.0001). Considering disease stage, a significantly higher number of cases were diagnosed as late stage 240 (93.4%) compared to early stage 17 (6.6%; p< 0.0001). For one patient, the disease stage could not be ascertained. There was no correlation between disease stage and age or gender of the patients.

**Table 1:** Demographic and baseline diagnostic characteristics of HAT patients.

Variable	Males	Females	Total	p-value
**Age (years), mean ±SEM**	28.3 ± 1.9	29.0 ± 1.5		0.756
**Age group (years)**				
<18	52 (57.8%)	38 (42.2%)	90 (35.3%)^a^	0.011
18–35	31 (35.2%)	57 (64.8%)	88 (34.5%)	
36–53	20 (44.4%)	25 (55.6%)	45 (17.6%)	
>54	19 (59.3%)	13 (40.6%)	32 (12.5%)	
**Disease Stage**				
Early	8 (47.1%)	9 (52.9%)	17 (6.6%)	<0.0001
Late	116 (48.3%)	124 (51.7%)	240 (93.4%)^b^	
**Trypanosomes in CSF**	17 (48.6%)	16 (45.7%)	35 (43.2%)	0.257
**CSF WBC counts**				
<5	12 (52.2%)	11 (47.8%)	23 (10.2%)	0.131
6–19	39 (40.6%)	57 (59.4%)	96 (42.7%)	
20–99	52 (57.8%)	38 (42.2%)	90 (40%)	
>100	8 (50%)	8 (50%)	16 (7.1%)	
**Area of residence**				
Kaberamaido	76 (46.1%)	89 (53.9%)	165 (69%)^c^	<0.0001
Dokolo	30 (55.6%)	24 (44.4%)	54 (22.6%)	
Alebtong	6 (46.2%)	7 (53.8%)	13 (5.4%)	
Lira	0	1 (100%)	1 (0.4%)	
Soroti	3 (60%)	2 (40%)	5 (2.1%)	
**Kole**	**0**	**1 (100%)**	**1 (0.4%)**	

CSF: cerebrospinal fluid, WBC: white blood cell

^a^significantly higher in patients <18 years

^b^significantly higher than early stage

^c^significantly higher in Kaberamaido

### Clinical symptoms and signs

Among the early stage signs, headache and fever were commonly observed (66.1% and 64.5% respectively; Table 2). Fever was significantly (p = 0.045) more common in patients under 18 years (S1 Table). A chancre was observed in 2.7% of the patients and was significantly more common in female patients (p = 0.031). Diarrhea (26.3%) and abdominal pain (26.3%) were significantly more common among female patients (p = 0.048 and p = 0.002 respectively). For patients below 18 years, ascites (p = 0.08) and cough (p = 0.016) were common below 4years while headache (p = 0.004) in children between 10–14 years. Among the neurological symptoms, somnolence (25.2%) and mental confusion (12.4%) were commonly observed. Tremors (7.4%), stiff neck (10.2%) and visual impairment (4.1%) were more common among female patients. Neurological signs were common in patients below 35 years with mental confusion being significantly more associated with patients below 18 years (p = 0.039). Eighteen patients (8.3%) of the patients were unconscious upon admission. There was no significant association between clinical symptoms and the WBC counts. Due to the limited number of early stage patients, it was not possible to meaningfully compare clinical symptoms of the 2 disease stages. However, 4 (26.7%) and 1 (6.7%) of early stage patients presented with late stage signs of sleep disorder and mental confusion respectively. Of these, 1 patient exhibiting mental confusion subsequently relapsed after suramin treatment, pointing to inaccurate staging.

**Table 2:** HAT clinical signs and symptoms by gender at time of admission.

Clinical characteristic	Males	Females	Total	p-value
Headache	68	79	147 (66.1%)	0.89
Fever	62	80	142 (64.5%)	0.323
General malaise	41	43	84 (38.4%)	0.578
Abdominal discomfort	16	41	57 (26.3%)	0.002^a^
Somnolence	22	33	55 (25.2%)	0.348
Edema	21	32	53 (24.7%)	0.34
Cough	21	28	49 (22.9%)	0.75
Vomiting	21	28	49 (22.6%)	0.63
Joint pains	17	20	37 (17.1%)	0.556
Anorexia	16	19	35 (16.1%)	0.556
Chest pain	12	19	31 (14.3%)	0.439
Body chills	9	18	27 (12.4%)	0.215
Mental confusion	16	11	27 (12.4%)	0.216
Diarrhea	6	17	23 (10.6%)	0.048^a^
Stiff neck	9	13	22 (10.2%)	0.656
Pruritus	9	10	19 (8.7%)	0.556
Loss of conciseness	10	8	18 (8.3%)	0.46
Tremors	7	10	17 (7.8%)	0.8
Splenomegaly	11	6	17 (7.8%)	0.13
Ascites	8	7	15 (6.9%)	0.6
Lymphadenopathy	6	9	15 (6.9%)	0.79
Back arch	7	7	14 (6.5%)	0.675
Wasting	7	6	13 (6.1%)	0.576
Jaundice	3	8	11 (4.9%)	0.23
Muscle pain	2	7	9 (4.2%)	0.184
Hepatomegaly	4	5	9 (4.1%)	0.597
Peri-orbital edema	3	6	9 (4.1%)	0.514
Visual impairment	1	5	6 (4.1%)	0.222
Dysuria	3	5	8 (3.7%)	0.729
Incontinence	3	4	7 (3.2%)	0.592
Restlessness	3	3	6 (2.8%)	0.835
Chancre	0	6	6 (2.7%)	0.031^a^
Paralysis	2	1	3 (1.3%)	0.597

^a^significantly higher in female patients

### Disease outcome

Disease duration was estimated from the patient interview data and considered the time when first HAT related symptoms were recognized to the time of hospitalization. The average onset of clinical signs was 2.2±2.3 (0.25–12) months showing significant individual variation in disease progression. Disease duration was significantly higher among mortality cases (3.1 ± 0.8 months; p< 0.0001) as compared to those discharged (2.1 ± 0.2 months). A significant proportion of patients, 68 (58.6%) reported onset of symptoms after one month and below with only 3 (2.6%) reporting symptoms at more than 8 months (p< 0.0001; Table 3). Both early (70%) and late (52.6%) stage patients had first symptoms within the past month or less. This points to a rapid progression to the neurological stage. Gender, age, WBC counts, district of origin and presence of trypanosomes in the CNS did not significantly affect disease duration. The average pre-treatment WBC count was 31.5±40cells/μl (3–320) while that after treatment was 5.0±6.7cells/μl (1–60). Patients with trypanosomes in the CNS had significantly higher (p = 0.003) WBC count 32.82 ± 6.0cells/μl compared with those with no CNS trypanosomes 15.52±2.3cells/μl. The mean hemoglobin levels were 8.24±1.96 g/dL (4.8–13.2). Blood transfusion was done in 7 (2.9%) of the patients with a mean hemoglobin of 6.88 ± 0.7 g/dL.

**Table 3:** HAT disease outcome.

Variable	Males	Females	Total	p-value
**Mortality**				
Early stage	0	0	0 (0%)	
Late stage	15 (55.6%)	12 (44.4%)	27 (10.5%)	0.424
**RE**	9 (47.4%)	10 (52.6%)	19 (7.9%)	0.919
**RE mortality**	4 (57.1%)	3 (42.9%)	7 (36.8%)	0.051
**Relapse**	6 (60%)	4 (40%)	10 (4%)	0.529
**Relapse time (Mean ± SEM)**	5.6±1.2	7.6±2		0.381
**Death after admission (days)**				
1–5	3	3	6	0.302
6–10	3	3	6	
>11	6	1	7	
**Onset of symptoms (months)**				
<1	32 (47.1%)	36 (52.9%)	68 (58.6%)	0.601
2–4	13 (52%)	12 (48%)	25 (21.6%)	
5–7	7 (35%)	13 (65%)	20 (17.2%)	
8–12	2 (66.7%)	1 (33.3%)	3 (2.6%)	
**Duration in hospital (days)(Mean ± SEM)**	30.2±1.3	30.2±1.8		0.995
**Hemoglobin (g/dL)(Mean ± SEM)**	8.0 ± 0.6	8.5 ± 0.5		0.581
**Blood transfusion**	2 (28.6%)	5 (72.4%)	7 (2.9%)	0.452

RE: Reactive Encephalopathy

After admission, 27 (10.5%) of the patients died in hospital (Table 3). The average period to death after admission was 10.8±7.3 (1–29) days. Reactive Encephalopathy (RE) was observed in 19 (7.9%) patients among these 7 died (36.8%). Death due to RE was on average after 4.2±0.8 melarsoprol intravenous injections. There was no association between cell counts, having trypanosomes in the CNS and mortality. Mortality rates were significantly associated with late stage signs like tremors (p = 0.03) and loss of consciousness (p< 0.0001) prior to admission. A relapse rate of 4% (10) was observed. Among the relapses, 7(70%) had been treated previously as early stage indicating potential miss-staging. A significant (p = 0.033) number of males relapsed in early stage, 6 (85.7%) with male patients having a 75% likelihood to relapse after early stage treatment. The average relapse time was 6.5+3.2 (2.5–12) months and did not significantly differ across gender, age, or disease stage.

### Prevalence of Co-infections

The overall prevalence of co-infections was 37.2% (90/242). Malaria was significantly more prevalent at 28.9% (p< 0.0001) followed by urinary tract infections (UTIs) recorded in 10 HAT patients (4.2%; S2 Table). Co-infections were present in 14.3% (12) of in-hospital deaths; malaria was common in this group 38.5%. Considering age-group, malaria was significantly more common in patients under 18 years (45.5%; p< 0.02) and was reported in 60% of the fatal cases in this age group. Within this age group, malaria was common in patients below 10 years. HIV and UTI co-infections were significantly associated with patients above 45 years (p< 0.004 and P< 0.007 respectively). A proportion of HAT cases (10.7%) were co-infected by 2 other diseases; of these, malaria and urinary tract infections (UTIs) were the more common (44%) followed by malaria and typhoid fever (22.2%).

### Effect of co-infections on disease outcome

Malaria and other co-infections did not significantly affect HAT clinical presentation and case fatality rates (S3 Table). Upon admission, HAT patients were treated with an average of 7.4±2.1 (5–14) different drug types (HAT treatment drugs inclusive). Co-infected patients received significantly more drug types compared to those diagnosed with HAT alone (p = 0.03; Table 4). The average hospital stay was 30.1±10.6 (10–68) days being significantly longer (p< 0.042) among HAT patients with co-infections (38.2±10.2). The average treatment cost (excluding HAT specific drugs) was estimated at USD 9.0±2.3 (3.8–12.3), with co-infected patients paying significantly higher for treatment (p = 0.002).

**Table 4:** Effect of co-infections on HAT disease outcome.

Variable	Co-infection	No co-infection	p-value
**Mortality**	12	14	0.199
**Trypanosomes in CSF**	7	25	0.144
**CSF WBC count**	28.6 ± 3.6	32.7 ± 3.7	0.476
**RE**	8	9	0.293
**Relapse**	3	7	0.756
**Death after admission (days)**			
1–5	1	5	0.412
6–10	2	3	
>11	4	3	
**Onset of symptoms (months)**			
<1	23	44	0.601
2–4	10	14	
5–7	6	14	
8–12	0	3	
**Drugs types received (Mean ± SEM)**	10±1.5	7.0±2.2	0.03^a^
**Duration in hospital (days) (Mean ± SEM)**	38.2±10.2	30±9.5	0.042^a^
**Hemoglobin (g/dL) (Mean ± SEM)**	7.6 ± 0.4	8.4± 0.6	0.34
**Blood transfusion**	2	5	0.763
Hospital dues paid ($)	**8.4±2.2**	**4.2±1.9**	**0.002^a^**

RE: Reactive Encephalopathy, CSF: cerebrospinal fluid, WBC: white blood cell

^a^significantly higher in HAT patients with co-infections

## Discussion

Based on retrospective data from patient record at Lwala hospital, we describe the clinical presentation, disease outcome and co-morbidities associated with sleeping sickness in north eastern Uganda. The data shows that a significant number of patients (p< 0.0211) presented at the hospital in 2005 as compared to subsequent years. The spread of sleeping sickness into this region is attributed to the importation of *T*. *b*. *rhodesiense* infected cattle [16,17] from the Busoga region (SE Uganda). HAT outbreaks started occurring in this region from about 1998. The subsequent reduction in sleeping sickness cases from 2006 may be attributed to projects involved in tsetse vector control and mass treatment of cattle [18].

A significant number of cases were below 18 years (p< 0.0001) with male children being significantly more common. This age variation in disease prevalence might be attributed to age specific activities [19,20]. In north eastern Uganda, young males are involved in grazing animals and in so doing are exposed to infected tsetse flies. Similarly, previous studies have reported sleeping sickness to be highly prevalent among the most productive age groups [21]. Unlike previous reports [20,22], gender did not significantly influence the number of cases, indicating a probably shared role of farming activities.

The clinical profiles followed the usual HAT presentation with a wide spectrum of individual variation in disease presentation. Unspecific symptoms like headache (66.1%) and fever (64.5%) were commonly observed. Headache is a common sign that has been reported in other studies [4,22,23]. Similarly, the prevalence of fever in this study was higher than that reported among *T*. *b*. *gambiense* patients [24–26] and in previous *T*. *b*. *rhodesiense* disease studies [5,22]. Fever was significantly common in patients below 18 years. Other studies have reported fever to be common in children [24]. A chancre develops 5–15 days after a tsetse bite and is an indicator of recent infection in *T*. *b*. *rhodesiense* disease [5,8]. In this study, a chancre was reported in 2.7% of the cases, much lower than reported by MacLean et al. [5] among Ugandan patients. It is however higher than that reported in Malawi patients by the same author. This variation might be attributed to variation in parasite virulence or differential host response to disease [25,27].

Several studies have shown that the hallmark of second stage HAT disease is presence of neurological signs [22,28,29]. Neurological manifestations were observed in both late and early stage patients. Sleep disorders reported in this study were lower than reported in previous studies [4,5,22] and this could be attributed to possible variation in parasite strains involved. Distinguishing between early and late stage on the basis of neurological symptoms has been shown to be challenging [30]. In this study, 26.7% and 6.7% of early stage patients presented with late stage signs of sleep disorder and mental confusion respectively. Presence of neurological signs among early stage patients might be attributed to a rapid progression of disease in Ugandan patients before the parasites and or increased WBC counts can be demonstrated in CSF. This variation in disease spectrum has already been reported among African populations [31,32]. Recent genomic and immunological data has pointed to possible variation in parasite strains or variation in host immunological responses [31,33]. The average pre-treatment WBC count was 31.5±40 cells/μl indicating that a large number of patients had progressed to late stage disease on reporting to hospital.

In this study, we report a 10.5% fatality rate that is in the same range as reported by MacLean et al. [5] among Tororo (Uganda) HAT patients (11%) but higher than that reported among Soroti (Uganda) patients (6%) and by Pepin et al. [10] among *T*. *b*. *gambiense* patients (5.7%). Reactive Encephalopathy was observed in 7.9% of the patients with a significant fatality rate of 36.8%. Our findings are in line with previous studies reporting RE to occur in 5–15% of the patients with a fatality rate of 10–50% [23,26,34].

We report a relapse rate of 4% (10/253) with 7 (70%) of the cases relapsing after early stage suramin treatment. One of these patients had late stage symptoms, pointing to a possibility of miss-staging. Males had 75% likelihood to relapse after early stage treatment. Suramin the early stage drug has been shown to be stable with limited potential for resistance [35]. Hence early stage relapse might be attributed to a stage when parasites have just crossed the blood brain barrier or pointing to miss staging. In such a scenario when WBCs are yet to be elevated, the patient will be classified as early stage when the few trypanosomes in the CSF are missed by microscopy. A high relapse rate in male patients might be due to faster disease progression that can presently not be fully explained. Given this observation, one might argue for treatment of all early stage patients as late stage irrespective of the CSF findings, if a safe drug were available. Of the melarsoprol treated patients, 3 (1.25%) relapsed indicating potential drug failure. Resistance to melarsoprol has already been reported in previous studies [12,36], although hitherto not considered a big problem in *T*. *b*. *rhodesiense*. Based on the estimated duration of infection, average onset of clinical signs was 2.2±2.3 (0.25–12) months showing significant individual variation in disease progression. This study is in agreement with work by MacLean et al. [5] among Soroti (Uganda) patients in which late stage was estimated to occur in less than 2 months unlike in Malawi patients at 5 months after infection.

We determined the prevalence of co-infections among HAT cases and their effect on HAT disease outcome. Our data shows that HAT is common in regions that are endemic for other diseases. The overall prevalence of co-infections was 37.2% with malaria being significantly more prevalent. In comparison Kagira et al. [22] reported a malaria prevalence of 100% among *T*. *b*. *rhodesiense* HAT patients in Kenya. Kuepfer et al. [4] reported a prevalence of 79.7% in Tanzanian *T*. *b*. *rhodesiense* patients and 2.9% among Ugandan patients. It is important to keep track of these co-infections; studies in *T*. *b*. *gambiense* have shown that HIV might negatively affect treatment outcome [37,38]. Furthermore studies have shown that helminthes negatively affect HIV/AIDS, malaria, and TB outcome [39,40]. However, like in other studies [4,5,22], malaria and other co-infections apparently did not significantly affect HAT clinical presentation and case fatality rates. We investigated the burden of co-infections on the patient. Our study has indicated that HAT patients harboring co-infections received significantly more drug types so as to clear all the infections. Toxicity effects that those drug combinations impose on the patients have not been researched but when used alone, suramin and melarsoprol have been shown to be considerably toxic [41]. Furthermore co-infected individuals had longer hospital admissions coupled with higher treatment costs. HAT specific treatment is offered by WHO therefore the extra cost is as a result of payment of the extra drugs and hospital bed space. The cost incurred is bound to be higher than reported in this study since calculations do not include indirect costs to the patients house hold and hospital attendance that have been found to be significant elsewhere [42,43]. Given the retrospective design of this study, it’s possible that a number of co-infections could have been missed. With respect to the above, more research efforts are required to understand the complex interaction of co-infections for neglected tropical disease [13].

In conclusion, this retrospective study has shown a wide spectrum of individual disease presentation with some patients suffering severe disease and progressing faster than others. It would be interesting to determine the host and parasite factors responsible for the individual clinical diversity. Our study further shows that malaria is the most common co-infection associated with sleeping sickness in north eastern Uganda. The prevalence of co-infections is bound to be higher than reported since 17.1% of the patients had been treated of other diseases before HAT suspicion and eventual diagnosis. It would be of paramount importance for HAT control programs to target multiple diseases in an integrated approach. Our study has further shown that though co-infections might not significantly influence HAT clinical outcome, they significantly increase the drug toxicity burden on the patient leading to extended hospital stay that incurs an extra cost to the patient and care taker. It would hence be important to investigate the complex interactions and the impact these co-infections might have on immunity. Such research would enable us understand the mechanisms underlying protection or design more effective therapeutic strategies.

## Supporting Information

S1 Table HAT clinical signs and symptoms by age category at time of admission. (DOCX)

S2 Table HAT co-infections categorized by age. (DOCX)

S3 Table HAT clinical signs and symptoms of malaria co-infected patients. (DOCX)
