# Supplementary material for: Clinical Profiles, Disease Outcome and Co-Morbidities among T. b. rhodesiense Sleeping Sickness Patients in Uganda
Source: PLoS One. 2015 Feb 26;10(2):e0118370. doi: 10.1371/journal.pone.0118370 (PMC4342333; doi:10.1371/journal.pone.0118370)
Supplement: S1 Table — (DOCX) [file pone.0118370.s001.docx]

**S1 Table**. HAT clinical signs and symptoms by age category at time of admission

| Clinical characteristic | <18 Years | 18-35 | 36-53 | >54 | p-value |
| --- | --- | --- | --- | --- | --- |
| Headache | 41 | 58 | 25 | 20 | 0.155 |
| Fever | 55 | 54 | 20 | 12 | 0.045^a^ |
| General malaise | 22 | 34 | 15 | 12 | 0.379 |
| Abdominal discomfort | 17 | 23 | 8 | 8 | 0.746 |
| Somnolence | 26 | 16 | 8 | 4 | 0.078 |
| Edema | 14 | 24 | 5 | 10 | 0.05 |
| Cough | 16 | 23 | 3 | 7 | 0.079 |
| Vomiting | 17 | 16 | 7 | 9 | 0.365 |
| Joint pains | 3 | 4 | 2 | 0 | 0.856 |
| Anorexia | 8 | 13 | 6 | 8 | 0.14 |
| Chest pain | 9 | 9 | 8 | 4 | 0.447 |
| Body chills | 8 | 15 | 11 | 3 | 0.108 |
| Mental confusion | 11 | 5 | 8 | 1 | 0.039^a^ |
| Diarrhea | 9 | 9 | 1 | 4 | 0.27 |
| Stiff neck | 10 | 8 | 3 | 1 | 0.581 |
| Pruritus | 9 | 9 | 0 | 1 | 0.08 |
| Loss of conciseness | 4 | 9 | 5 | 0 | 0.166 |
| Tremors | 8 | 8 | 0 | 1 | 0.135 |
| Splenomegaly | 9 | 5 | 1 | 2 | 0.295 |
| Ascites | 5 | 6 | 1 | 3 | 0.551 |
| Lymphadenopathy | 6 | 6 | 1 | 2 | 0.756 |
| Back arch | 1 | 6 | 4 | 3 | 0.109 |
| Wasting | 6 | 3 | 3 | 1 | 0.579 |
| Jaundice | 2 | 4 | 3 | 2 | 0.529 |
| Muscle pain | 5 | 15 | 3 | 3 | 0.168 |
| Hepatomegaly | 3 | 5 | 1 | 0 | 0.748 |
| Peri-orbital edema | 3 | 5 | 1 | 0 | 0.749 |
| Visual impairment | 0 | 4 | 1 | 0 | 0.231 |
| Dysuria | 4 | 2 | 1 | 1 | 0.741 |
| Incontinence | 2 | 4 | 0 | 1 | 0.609 |
| Restlessness | 3 | 0 | 1 | 0 | 0.207 |
| Chancre | 1 | 3 | 1 | 1 | 0.823 |
| Paralysis | 2 | 1 | 0 | 0 | 0.732 |

^a^significantly higher in patients less than 18 years
