# Supplementary material for: Clinical Profiles, Disease Outcome and Co-Morbidities among T. b. rhodesiense Sleeping Sickness Patients in Uganda
Source: PLoS One. 2015 Feb 26;10(2):e0118370. doi: 10.1371/journal.pone.0118370 (PMC4342333; doi:10.1371/journal.pone.0118370)
Supplement: S2 Table — (DOCX) [file pone.0118370.s002.docx]

**S2 Table**. HAT co-infections categorized by age.

| **Co-infections** | **<18 Years** | **18-35** | **36-53** | **>54** | **Total** | **p-value** |
| --- | --- | --- | --- | --- | --- | --- |
| Malaria | 33 | 21 | 10 | 6 | 70 (28.9%) | 0.02^a^ |
| HIV | 0 | 0 | 3 | 0 | 3 (1.2%) | 0.006^b^ |
| Tuberculosis | 0 | 1 | 1 | 0 | 2 (0.8%) | 0.614 |
| Typhoid | 0 | 4 | 0 | 0 | 4 91.7%) | 0.126 |
| Urinary tract infections | 0 | 1 | 5 | 4 | 10 (4.1%) | 0.021^b^ |
| Cryptococcosis | 1 | 0 | 0 | 0 | 1 (0.4%) | 0.633 |
| Amebiasis | 1 | 0 | 0 | 0 | 1 (0.4%) | 0.625 |
| Streptococcal meningitis | 1 | 0 | 0 | 0 | 1 (0.4%) | 0.625 |
| Total | 38 (42.2%) | 24(26.7%) | 18 (20%) | 8 (8.9%) | 90 (37.2%) | <0.0001^a^ |
|  |  |  |  |  |  |  |

^a^significantly higher in patients below 18 years

^b^significantly higher in patients above 35 years
