# Supplementary material for: Clinical Profiles, Disease Outcome and Co-Morbidities among T. b. rhodesiense Sleeping Sickness Patients in Uganda
Source: PLoS One. 2015 Feb 26;10(2):e0118370. doi: 10.1371/journal.pone.0118370 (PMC4342333; doi:10.1371/journal.pone.0118370)
Supplement: S3 Table — (DOCX) [file pone.0118370.s003.docx]

**S3 Table**. HAT clinical signs and symptoms of malaria co-infected patients

| Clinical characteristic | Malaria positive | Malaria negative | p-value |
| --- | --- | --- | --- |
| Fever | 40 (28.4%) | 101 (71.6%) | 0.539 |
| Chancre | 1 (16.7%) | 5 (83.3%) | 0.673 |
| Headache | 47 (32.4%) | 98 (67.6%) | 0.274 |
| Edema | 13 (24.5%) | 40 (75.5%) | 0.389 |
| Lymphadenopathy | 6 (40%) | 9 (60%) | 0.386 |
| Splenomegaly | 5 (29.4%) | 12 (70.6%) | 0.984 |
| Hepatomegaly | 1 (11.1%) | 8 (88.9%) | 0.287 |
| Ascites | 4 (26.7%) | 11 (73.3%) | 0.794 |
| Tremors | 6 (35.3%) | 11 (64.7%) | 0.588 |
| Loss of conciseness | 2 (11.1%) | 16 (88.9%) | 0.104 |
| Cough | 15 (31.3%) | 33 (68.7%) | 0.858 |
| Vomiting | 15 (30.6%) | 34 (69.4%) | 0.86 |
| Diarrhea | 9 (39.1%) | 14 (60.9%) | 0.335 |
| Abdominal discomfort | 20 (35.7%) | 36 (64.3%) | 0.308 |
| Somnolence | 20 (34.5%) | 38 (65.5%) | 0.237 |
| Pruritus | 7 (38.9%) | 11 (61.1%) | 0.421 |
| General malaise | 24 (28.9%) | 59 (71.1%) | 0.879 |
| Chest pain | 7 (22.6%) | 24 (77.4%) | 0.403 |
| Stiff neck | 6 (27.3%) | 16 (72.7%) | 0.787 |
| Mental confusion | 8 (47.1%) | 9 (52.9%) | 0.987 |
| Anorexia | 11 (31.4%) | 24 (68.6%) | 0.841 |
| Muscle pain | 3 (33.3%) | 6 (66.7%) | 0.723 |
| Body chills | 6 (22.2%) | 21 (77.8%) | 0.5 |
| Joint pains | 13 (28.3%) | 23 (71.7%) | 0.424 |
| Incontinence | 3 (42.9%) | 4 (57.1%) | 0.421 |
| Dysuria | 2 (25%) | 6 (75%) | 0.785 |
| Restlessness | 4 (66.7%) | 2 (33.3%) | 0.062 |
| Back arch | 5 (38.5%) | 8 (61.5%) | 0.669 |
| Jaundice | 2 (18.2%) | 9 (81.8%) | 0.52 |
| Peri-orbital edema | 2 (22.2%) | 7 (77.8%) | 0.64 |
| Paralysis | 0 | 3 (100%) | 0.561 |
| Visual impairment | 2 (33.3%) | 4 (66.7%) | 0.825 |
| Wasting | 2 (15.4%) | 11 (84.6%) | 0.353 |
|  |  |  |  |
